# Lipid metabolism as a central driver of immune remodeling and therapeutic vulnerability in metastatic colorectal cancer

**DOI:** 10.1186/s12944-026-02956-9

**Published:** 2026-04-30

**Authors:** Yuru Shang, Qingxi Yang, Runlin Zhang, Weiguo Xu, A. M. Abd El-Aty, Yu Gao, Tianbao Wang

**Affiliations:** 1 Department of Gastrointestinal Surgery South China Hospital, Medical School & Guangdong Key Laboratory for Biomedical Measurements and Ultrasound Imaging, School of Biomedical Engineering, National-Regional Key Technology Engineering Laboratory for Medical Ultrasound, Shenzhen University, Shenzhen University Medical School, Shenzhen, 518060 China; 2 Department of Oncology, Shandong Provincial Taishan Hospital, Taian, 271000 China; 3 Institute of Pharmacy (Institute of TCM Health Industrial Technology), Shandong University of Traditional Chinese Medicine, Jinan, 250355 China; 4 Institute of Alternative Medicine, Leverkusen, 5651373 Germany; 5 Department of Medical Pharmacology, Medical Faculty, Ataturk University, Erzurum, 25240 Turkey; 6 Department of Pharmacology, Faculty of Veterinary Medicine, Cairo University, Giza, 12211 Egypt; 7 Department of Oncology, Shanxi Provincial Academy of Traditional Chinese Medicine, Taiyuan, 030000 China

**Keywords:** Lipid metabolism, Colorectal cancer, Tumor immune microenvironment

## Abstract

Dysregulated lipid metabolism has emerged as a defining hallmark of colorectal cancer (CRC) progression, particularly in metastatic disease, where metabolic adaptation and immune evasion are tightly interconnected, as demonstrated in both murine models and human studies. Increasing evidence has demonstrated that alterations in lipid synthesis, uptake, transport, and oxidation not only sustain tumor bioenergetics but also actively remodel the tumor immune microenvironment. Key lipid metabolic regulators—including FASN, SREBP signaling, CD36-mediated lipid uptake, cholesterol metabolism, and fatty acid oxidation—coordinate oncogenic signaling and promote immunosuppressive states characterized by T-cell exhaustion, macrophage polarization, and ferroptosis resistance, on the basis largely of correlative and preclinical evidence. Recent advances in multiomics technologies, including single-cell and spatial transcriptomics, metabolomics, and lipidomics, have enabled high-resolution mapping of lipid-dependent immune niches within metastatic CRC (mCRC) lesions. These approaches reveal lipid metabolism as a central organizer of tumor–immune interactions and identify previously unrecognized metabolic vulnerabilities. In this review, we integrate current knowledge on lipid metabolic reprogramming in CRC with emerging multiomics insights, highlighting the mechanisms linking lipid metabolism, ferroptosis, gut microbiota interactions, and immune remodeling. We further discuss therapeutic strategies targeting lipid metabolic pathways and their potential synergy with immunotherapy. Collectively, the results of this work suggest that understanding lipid metabolism is a unifying framework for understanding mCRC biology and developing metabolism-guided therapeutic interventions.

## Introduction

 Colorectal cancer (CRC) originates from the colon or rectum and is the third most common cancer globally. It is also the second leading cause of cancer-related deaths and is particularly prevalent in developed regions [1–3]. In 2020, there were more than 1.9 million new cases and approximately 900,000 deaths due to CRC, and the incidence continues to increase [4]. The accumulation of driver genetic mutations, such as those in *APC*, *MLH1*, and *KRAS*, is a leading cause of CRC [5–7]. CRC often begins as a small polyp, most of which is benign and can be completely excised [2]. However, some polyps can become malignant and develop into carcinomas over time. As a result, many patients are not diagnosed until an advanced stage is reached because of the absence of polyp development and the lack of obvious symptoms [8, 9]. The urgency of developing novel therapeutic methods for CRC cannot be overlooked.

In addition to genetic alterations, metabolic reprogramming has emerged as a hallmark of CRC progression, with lipid metabolism occupying a particularly prominent position. Lipid metabolic processes—including de novo lipogenesis, lipid uptake, oxidation, transport, and storage—are frequently dysregulated during CRC development [10–12]. As tumors progress toward invasive and metastatic disease, lipid metabolism reprogramming extends beyond cancer cells and actively reshapes the tumor microenvironment (TME). Increased fatty acid synthesis and uptake in both tumor cells and immunosuppressive stromal and immune populations promote immune tolerance, suppress cytotoxic T-cell activity, and facilitate immune escape, thereby enabling sustained tumor growth and dissemination [10, 13, 14]. Importantly, accumulating evidence indicates that the tumor microenvironment of mCRC lesions is fundamentally distinct from that of primary tumors. Metastatic sites such as the liver and lung impose unique metabolic and immunological constraints, shaping organ-specific lipid utilization patterns and immune cell adaptations. These differences critically influence immune surveillance, ferroptosis susceptibility, and therapeutic responsiveness, suggesting that lipid metabolism serves as a central coordinator of immune remodeling during CRC metastasis. However, conventional single-layer analytical approaches have been insufficient to capture the spatial, temporal, and cellular heterogeneity of lipid‒immune interactions in mCRC.

Recent advances in multiomics technologies, including bulk and single-cell transcriptomics, spatial transcriptomics, metabolomics, and lipidomics, have provided unprecedented resolution for dissecting the complex interplay between lipid metabolic programs and immune cell function within the TME [15, 16]. These integrative approaches have revealed that lipid metabolic programs are selectively activated in specific immune cell subsets—such as regulatory T cells, tumor-associated macrophages, and exhausted CD8⁺ T cells—and are spatially organized within the immunosuppressive niches of metastatic tumors [17, 18]. Notably, lipid metabolism is particularly well suited for multiomics interrogation, as lipid-driven phenotypes are governed by multilayered regulation spanning gene expression, enzymatic activity, metabolite availability, and spatial compartmentalization.

In this review, we summarized key lipid metabolism regulators and signaling pathways involved in CRC, with a focus on fatty acid and cholesterol metabolism. We highlighted how lipid metabolic reprogramming intersects with immune cell function and tumor microenvironment remodeling during CRC progression and metastasis. In addition, we discussed the roles of ferroptosis and the gut microbiota in shaping lipid metabolism-dependent tumor behavior. Finally, we reviewed recent advances in therapeutic strategies targeting lipid metabolism, particularly in combination with immunotherapy, aiming to provide a comprehensive, multiomics-informed framework for understanding lipid metabolism–driven immune remodeling and for developing innovative treatment strategies for advanced CRC.

## Lipid metabolic regulators in CRC

**Fig. 1:**
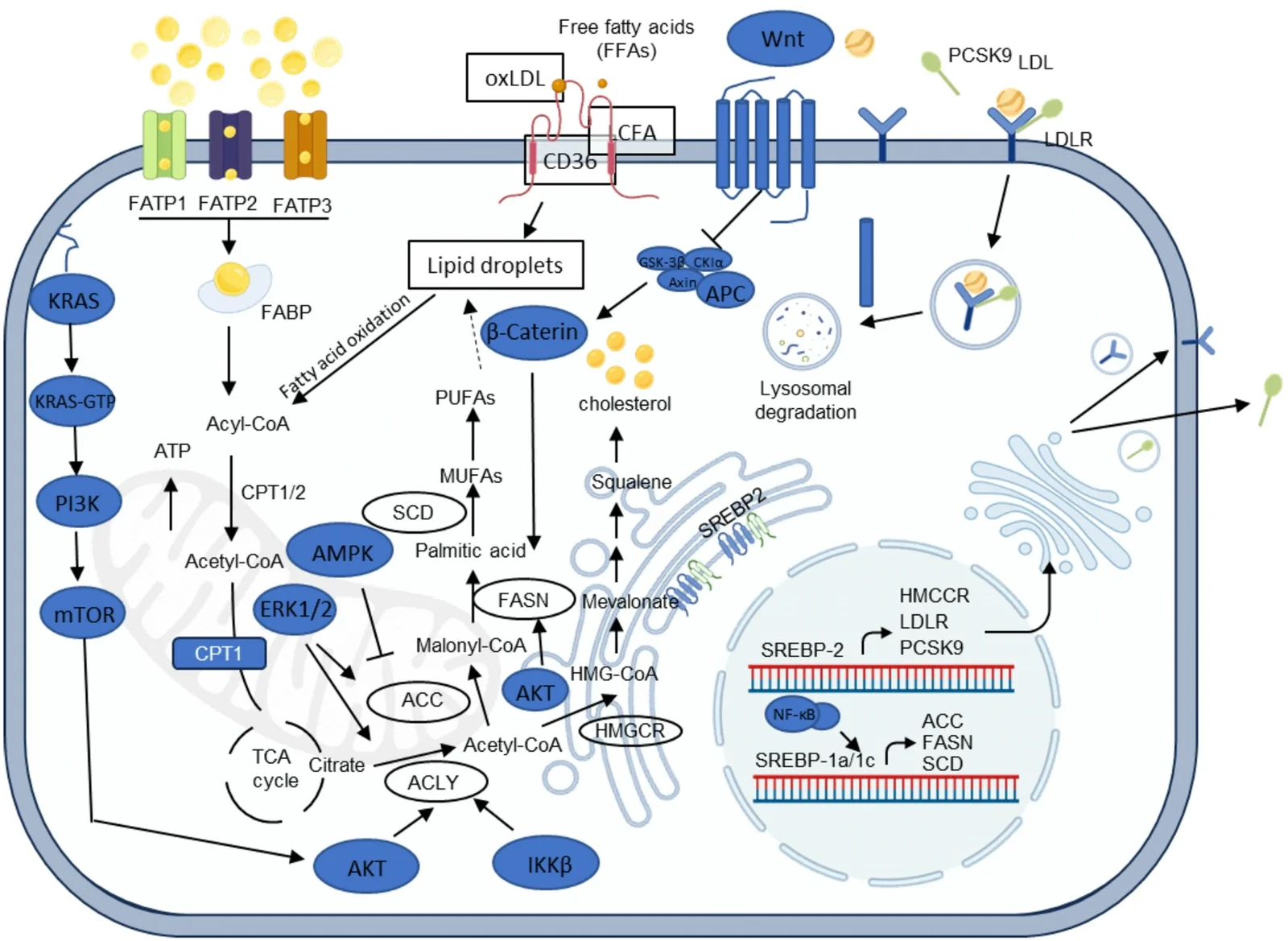
Lipid metabolic reprogramming and its interaction with oncogenic signaling in cancer. This schematic diagram illustrates the reprogramming of lipid metabolism in cancer cells and its interactions with oncogenic signaling pathways. Extracellular fatty acids (FFAs) and oxidized low-density lipoprotein (oxLDL) are taken up by transporters such as FATP, CD36, FABP, and LDLR and converted into acyl-CoA for β-oxidation or storage in lipid droplets. De novo lipid synthesis is driven by ACLY, ACC, and FASN, whereas cholesterol synthesis occurs via the HMGCR-mediated mevalonate pathway. These processes are regulated primarily by SREBP-1 and SREBP-2. Oncogenic pathways, including the KRAS-PI3K-AKT-mTOR and Wnt/β-catenin signaling pathways, promote lipid synthesis and metabolic adaptation, whereas AMPK inhibits lipidogenesis by targeting ACC. Furthermore, NF-κB synergistically enhances the expression of lipid metabolism-related genes with SREBP. These pathways collectively constitute a metabolic signaling network that supports tumor growth and progression

CRC involves multiple enzymes and proteins that drive dysfunctional lipid metabolism, creating favorable conditions for rapid cell division and growth. These regulators do not work in isolation; they work together or interact to provide essential lipid building blocks and energy to rapidly proliferating CRC cells (Fig. 1). The key regulators of lipid metabolism in CRC tissue include fatty acid synthase (FASN) and acetyl-CoA carboxylase (ACC), which initiate fatty acid synthesis; HMG-CoA reductase and PCSK9, which mediate cholesterol synthesis and regulation; and CD36, along with various lipid transport proteins, which facilitate lipid transport and utilization. In addition, we briefly summarize the roles of CPT1, SREBPs, and SCD1 in lipid metabolism and CRC development. This section focuses on lipid metabolism regulators that have been extensively studied in CRC and have important pathogenic roles. Understanding these regulators and their roles in lipid metabolism is indispensable for drug development and disease treatment.

### FASN and ACC

FASN and ACC are two vital enzymes that initiate fatty acid synthesis, both of which are frequently upregulated during CRC development [10, 19, 20]. FASN catalyzes the formation of palmitate from acetyl-CoA and malonyl-CoA [21, 22]. ACC converts acetyl-CoA to malonyl-CoA, the initial step in fatty acid synthesis [23]. Sufficient lipid synthesis ensures an adequate energy supply, maintaining tumor growth and metastasis. The overexpression of FASN and ACC is positively related to poor prognosis and increased metastasis in CRC [24–27]. Recently, FASN and ACC were reported to be upregulated by novel mechanisms in CRC. ACC1 transcription was significantly elevated because of the binding between circCAPRIN1 and STAT2. circCAPRIN1, a circular RNA, is associated with poor prognosis in CRC patients. This binding leads to increased fatty acid synthesis and utilization [28]. Recent research has revealed that the long noncoding RNA (lncRNA) POU6F2-AS1 is frequently expressed in CRC and is associated with poor prognosis. POU6F2-AS1 reprograms fatty acid metabolism in CRC cells by upregulating FASN expression, thereby increasing de novo lipid synthesis and promoting tumor cell proliferation. The mechanism involves m6A modification mediated by METTL3, which increases the expression of POU6F2-AS1 and subsequently promotes the transcription of FASN [24]. Conversely, a decreased level of FASN can partially suppress colorectal cancer progression. RA-XII, a bicyclic hexapeptidic glucoside isolated from *rubia yunnanensis*, has been shown to effectively reduce fatty acid levels by blocking SREBP and downregulating FASN and SCD1 [29]. RA-XII exerts its antitumor activity in CRC cells by inhibiting protective autophagy and activating the Akt/mTOR pathway [30]. It suppresses autophagy through regulating several signaling pathways, including NF-κB signaling, enhancing the cytotoxicity of RA-XII and increasing the susceptibility of CRC cells to other treatments. This significantly arrests colorectal carcinoma growth and metastasis [31]. In conclusion, FASN and ACC play indispensable roles in promoting CRC development by driving de novo lipogenesis, and their overexpression is consistently associated with poor prognosis, increased metastasis, and resistance to therapy.

### HMG-CoA reductase

Cholesterol synthesis depends on HMG-CoA reductase (HMGCR), which catalyzes the synthesis of mevalonic acid (MVA), an important component in cholesterol production [32, 33]. Increased cholesterol can maintain the biological activities of tumor cells, such as maintaining membrane integrity. High amounts of cholesterol can be stored in lipid rafts in the cell membrane and are involved in signaling pathways that support tumor growth and survival [34, 35]. Moreover, excessive cholesterol often contributes to an immunosuppressive environment in CRC, promoting immune evasion. In normal tissue, negative feedback regulation occurs between HMG-CoA and cholesterol [36]. When cholesterol levels are high, the activity of HMGCR is restricted to prevent extra cholesterol production. In addition, SREBPs play a positive role in this feedback mechanism. When cellular cholesterol levels are below the threshold, SREBPs are activated and translocated to the nucleus to accelerate the transcription of HMGCR and other genes involved in cholesterol synthesis [37, 38]. However, in cancerous tissue, HMG-CoA is frequently upregulated to increase the biosynthesis of cholesterol [39, 40]. Clinically, HMG-CoA expression is positively correlated with favorable colorectal tumor characteristics and poor prognosis [39, 41], indicating that it is a hotspot target in clinical research. Statins reduce cholesterol levels by competitively inhibiting HMGCR and are well established for cardiovascular disease management. Their role in CRC therapy remains investigational and controversial: while some retrospective cohort studies suggest a modest association with reduced CRC risk or improved survival, randomized clinical trials have not demonstrated a consistent benefit, and statins are not approved for CRC treatment [42–44]. Some groups have shown that statin treatment significantly blocks the growth of CRC cells through different mechanisms, and we will introduce more details on statin therapy later. In addition to fatty acid synthesis, tumor cells are highly dependent on the uptake of exogenous lipids to meet their enormous demands, which is mediated mainly by transporter proteins such as CD36.

### CD36

CD36 (fatty acid translocase) is a multifunctional scavenger receptor that promotes the transport and utilization of lipids [45]. CD36 is part of the class B scavenger receptor family and binds various ligands, such as oxidized low-density lipoprotein (oxLDL) and long-chain fatty acids (LCFAs) [46, 47]. This multifunctional protein is involved in almost all steps of lipid metabolism. Recent studies have shown that CD36 not only participates in lipid transport but also plays roles in immune microenvironment remodeling, ferroptosis, and tumor metastasis [48–50]. For example, CD36 is upregulated in M2-type macrophages in CRC, which is correlated with a protumor phenotype in clinical specimens; causal evidence from murine models has demonstrated that CD36-mediated lipid uptake in macrophages actively promotes immunosuppressive polarization [51]. CD36 preferentially transports lipid-rich vesicles into macrophages, fuelling them and creating an immunosuppressive microenvironment [51]. In intratumoral Treg cells, CD36 expression is often upregulated to increase their ability to utilize fatty acids, which is important for supporting their survival in nutrient-deprived and lactic acid-rich environments [52]. CD36 was recently shown to promote ferroptosis in tumor-infiltrating CD8⁺ T cells through a distinct molecular mechanism: CD36-mediated uptake of oxidized low-density lipoprotein (oxLDL) and long-chain polyunsaturated fatty acids elevates intracellular lipid peroxide levels, overwhelming GPX4-dependent antioxidant defense and triggering ferroptotic death. This process is further amplified by the lipid-rich TME, where CD36 upregulation on CD8⁺ T cells is correlated with increased expression of the exhaustion markers PD-1, TIM-3, and LAG-3 [53]. Collectively, these findings identify CD36-driven lipid peroxidation as a mechanistic link between exogenous lipid availability, ferroptosis susceptibility, and T-cell dysfunction in CRC. High expression of CD36 is closely related to tumor metastasis and evasion because it promotes epithelial‒mesenchymal transition (EMT) [54]. In CRC, CD36 upregulation leads to increased expression of matrix metalloproteinase 28 (MMP28), which degrades extracellular matrix (ECM) components. MMP28 further cleaves E-cadherin, resulting in the loss of cell adhesion and promoting EMT [55]. Accumulating evidence has demonstrated that CD36 plays a cornerstone role in lipid metabolism and tumorigenesis, making it a research hotspot in tumor therapy and lipid metabolism diseases.

### Lipid transport proteins

Lipid transport proteins include mainly fatty acid and cholesterol transport proteins that participate in lipid transport, uptake, and utilization, such as fatty acid binding proteins (FABPs) and fatty acid transport proteins (FATPs). FABPs are structurally conserved proteins with a water-filled binding pocket, allowing them to bind long-chain fatty acids [56]. FATPs are transmembrane proteins that facilitate the transport of fatty acids across the plasma membrane. Both proteins are upregulated during tumorigenesis [57, 58]. FABP family members, including FABP4 and FABP5, are reportedly involved in the metastasis and development of CRC [59–61]. High levels of FABP5 have been reported to promote CRC metastasis [62]. In CRC, DNA demethylation can upregulate FABP5 expression, which in turn activates NF-κB signaling through the production of proinflammatory cytokines, leading to CRC progression [61]. Similarly, many members of the FATP family promote tumorigenesis through immune regulation [58, 63, 64]. FATP2 contributes to the polarization and tolerance of neutrophils, leading to an immunosuppressive microenvironment [65]. FATP4 has been reported to bind to lipid droplet proteins to accelerate lipid transport and utilization in mitochondria, supporting tumorigenesis and the energy supply [66]. Interestingly, FATP5 overexpression in CRC is associated with a better prognosis—a counterintuitive finding given the general tumor-promoting role of lipid transport. A proposed mechanistic explanation is that FATP5, by preferentially channeling long-chain fatty acids toward mitochondrial β-oxidation rather than membrane biosynthesis or signaling lipid pools, may paradoxically limit the availability of lipid substrates for oncogenic signaling. Additionally, FATP5 modulates cell cycle progression and proliferation capacity, suggesting a tumor-suppressive function in specific CRC contexts; however, direct mechanistic evidence remains limited and warrants further investigation [67].

### LDLRs and PCSK9

Low-density lipoprotein receptors (LDLRs) are transmembrane proteins that facilitate the transport and utilization of cholesterol and are located predominantly on the surface of liver cells [68]. Cholesterol levels are tightly controlled by LDLR expression. It has recently been reported that LDLR is aberrantly regulated in CRC patients, especially in advanced stages [69, 70]. Additionally, recent data suggest that LDLR may be involved in the regulation of inflammation and affect the occurrence of tumors. Prostaglandin E2 (PGE2), a proinflammatory molecule, is positively correlated with increased cholesterol uptake, suggesting that LDLR-mediated cholesterol uptake can promote CRC progression by contributing to a proinflammatory environment [71, 72].

LDLR regulation and cholesterol metabolism are closely related to proprotein convertase subtilisin/kexin type 9 (PCSK9) signaling [73–75]. PCSK9 is a protein that regulates cholesterol metabolism. It binds to LDLRs in liver cells, leading to their degradation in lysosomes [76, 77]. This process leads to increased blood cholesterol concentrations. Therefore, PCSK9 inhibition can prevent the degradation of LDLRs, thereby increasing the number of receptors available to clear LDL cholesterol. PCSK9 has also been shown to promote CRC development. In *KRAS/APC*-mutant CRC, upregulation of PCSK9 is associated with poor prognosis. PCSK9 induces cholesterol biosynthesis and geranylgeranyl diphosphate (GGPP) upregulation, which activates KRAS/MEK/ERK signaling [78]. GGPP is an intermediate in the mevalonate pathway and plays a crucial role in cholesterol biosynthesis [79, 80]. Additionally, high expression of PCSK9 promotes the growth and metastasis of colorectal carcinoma through activating the EMT and the PI3K/Akt pathway [81]. Several preclinical studies have highlighted the potential of PCSK9 inhibition to remodel the immune microenvironment and enhance the efficacy of immune checkpoint inhibitors (ICIs) in murine models [82, 83]. However, clinical evidence supporting the use of PCSK9 inhibition as an oncological strategy in CRC remains limited to early-phase or exploratory data, and prospective trials are needed before this approach can be recommended clinically [82, 83]. PCSK9 inhibitors can enhance antitumor immune responses by increasing the clonal expansion of cytotoxic T cells into tumors and reducing the presence of regulatory T cells [84], suggesting that PCSK9 inhibitors might be novel therapies for CRC.

### CPT1

Carnitine palmitoyltransferase 1 (CPT1) is a mitochondrial enzyme that facilitates the transport and oxidation of fatty acids [85, 86]. CPT1 is upregulated in various tumors to support their increased energy demands [87–89]. CPT1-mediated fatty acid oxidation is involved in multiple aspects of tumorigenesis and development. The fatty acid oxidation enzyme CPT1A, an isoform of CPT1 mainly found in the liver, promotes macrophage polarization during fatty acid metabolism, accelerating colorectal carcinoma metastasis [90]. Recent data have revealed that CPT1 impairs ferroptosis and that intervention with CPT1 significantly increases ferroptosis and impedes colorectal tumor growth [91]. Acylcarnitine accumulation is an important characteristic of disordered fatty acid metabolism [92]. Treatment with the ferroptosis inducer SA-11, a derivative of stem alkaloid, significantly increases acylcarnitine production, which in turn increases CPT-1 levels. This upregulation of CPT-1 enhances ferroptosis in colorectal cancer cells by increasing ROS levels, leading to acylcarnitine buildup [91]. These findings suggest that targeting CPT-1 and acylcarnitine metabolism may offer novel therapeutic strategies for cancer treatment by enhancing ferroptosis induction. Fatty acid oxidation (FAO) is an important pathway in lipolytic metabolism, whereas the core transcriptional regulation of lipid synthesis is mediated by the SREBP family, which directly regulates the expression of key genes such as FASN, ACC, and SCD1.

### SREBPs and SCD

Sterol regulatory element-binding proteins (SREBPs) play crucial roles in lipid homeostasis by regulating the transcription and expression of genes related to lipid biosynthesis and uptake [93, 94]. SREBP-1a/1c drives fatty acid synthesis by upregulating FASN, ACC, and SCD, whereas SREBP-2 enhances cholesterol biosynthesis via HMGCR and LDLR [95]. Stearoyl-CoA desaturase-1 (SCD1) is an endoplasmic reticulum enzyme that catalyzes the biosynthesis of monounsaturated fatty acids (MUFAs), which are essential components of membrane lipids, signaling molecules, and energy storage. SCD-1, a specific isoform of the SCD enzyme, is the most widely studied and is crucial for regulating fat storage and energy balance [96, 97]. SCDs convert saturated fatty acids (SFAs) into monounsaturated fatty acids (MUFAs), reducing the availability of polyunsaturated fatty acids (PUFAs) prone to peroxidation [98]. SCD1 is often upregulated in CRC, and its activity is linked to tumor growth and survival [99]. In CRC, SCD1 contributes to the synthesis of monounsaturated fatty acids, supporting the rapid proliferation and metastasis of cancer cells. Recent studies have shown that SREBP-1 and SCD1 are interconnected in their regulation of lipid metabolism, with SREBP-1c directly enhancing the expression of SCD1 [100]. This relationship highlights the importance of lipid metabolism in CRC development and highlights potential therapeutic targets for disrupting lipid biosynthesis pathways to inhibit tumor growth. Notably, SCD1 and GPX4 also play critical roles in ferroptosis regulation, as discussed in detail in "Ferroptosis" section.

In summary, FASN and ACC drive fatty acid synthesis, HMGCR dominates cholesterol production, CD36 and FATPs/FABPs and LDLRs/PCSK9 regulate lipid uptake and transport, CPT1 promotes fatty acid oxidative energy production, and SREBPs/SCD1 act as the core transcriptional and modification nodes to integrate multiple signals. The synergistic dysregulation of these regulators constitutes the molecular basis for the reprogramming of lipid metabolism in CRC, laying the foundation for the subsequent activation of oncogenic signaling pathways, remodeling of the immune microenvironment, and the development of therapeutic targets.

### Lipid metabolism signaling in CRC

In addition to individual enzymatic regulators, lipid metabolism in CRC is governed by interconnected oncogenic signaling networks. The following subsections examine the principal lipid metabolic signaling axes—Wnt/β-catenin, PI3K/Akt, and AMPK—and their roles in coordinating lipid-driven tumor progression.

### Wnt/catenin signaling

Wnt/β-catenin signaling is the most well-known pathway in CRC cells. Mutations in the adenomatous polyposis coli (APC) gene are positively related to the incidence of polyps in the colon and rectum, which significantly increase the risk of CRC [101]. Aberrant activation of Wnt/β-catenin signaling is the most common signaling alteration in CRC [102]. Wnt/β-catenin signaling increases the transcription of FASN and fatty acid transport proteins, which are essential for lipid metabolism. This upregulation supports the increased demand for fatty acids in rapidly proliferating tumor cells [103]. Wnt/β-catenin signaling also interacts with other pathways, such as the PI3K/AKT/mTOR pathway, to further enhance lipid metabolism and tumorigenesis [104]. Blocking Wnt signaling can disrupt cholesterol homeostasis and inhibit CRC development [105]. A synthetic lethal phenotype resulting from Wnt signaling downregulation was observed in APC-mutated colonic cells following HMGCR inhibition treatment [106]. In colon carcinoma, Wnt/β-catenin signaling has been reported to accelerate fatty acid uptake and utilization, thereby facilitating tumor development. Recent studies have shown that the activation of Drp1 by fatty acids can lead to metabolic reprogramming in cancer cells. Moreover, this activation has been linked to the potentiation of Wnt/β-catenin signaling [107]. In conclusion, Wnt/β-catenin regulates CRC development through various pathways and might serve as a potential diagnostic and prognostic marker in CRC patients. Wnt signaling not only directly regulates lipid synthesis genes but also frequently synergizes with the PI3K/Akt pathway to amplify prosurvival and prometabolic signals.

### PI3K/Akt signaling

The PI3K/Akt signaling pathway plays important roles in the development and progression of CRC. This pathway is involved in various cellular processes, including cell growth, proliferation, survival, and lipid metabolism [108, 109]. This pathway regulates lipid metabolism primarily through the activation of SREBP and FASN, two key regulators of lipid biosynthesis [110]. Recently, PI3K/Akt was shown to inhibit ferroptosis. Activation of the PI3K-Akt-mTOR pathway initiates oncogenic signaling and induces tumorigenesis, which is partly mediated by ferroptosis resistance [111–113]. Researchers have reported that inhibiting the PI3K/Akt pathway sensitizes CRC cells to ferroptosis. Specifically, the use of a PI3K inhibitor in combination with the ferroptosis inducer RSL3 significantly increased lipid peroxidation and cell death [114]. Mechanistically, SREBP- and SCD1-mediated lipogenesis plays essential roles in this process [115]. In addition, upregulation of PI3K/Akt expression is positively related to enhanced lipid synthesis and increased expression of FASN, both of which significantly exacerbate CRC development and facilitate metastasis [116]. As a result, combining FASN inhibitors with PI3K inhibitors can alleviate tumor development [117]. The PI3K/Akt-mTOR axis is a potent driver of lipid synthesis and is also a key pathway for inhibiting iron death and maintaining tumor cell survival. On the other hand, AMPK often acts as an energy sensor and is antagonistic to PI3K/Akt.

### AMPK signaling

The AMP-activated protein kinase (AMPK) signaling pathway orchestrates many biological events, including energy metabolism, cell proliferation, autophagy, and cell polarization [118, 119]. AMPK acts as an energy sensor to maintain energy metabolism homeostasis. However, in tumor cells, AMPK activity is often restricted to accelerate lipogenesis and provide sufficient energy for tumor cell growth [120]. In a high-fat diet, AMPK can coordinate with the sonic hedgehog pathway to trigger colorectal tumorigenesis. This interaction promotes the proliferation and survival of cancer cells by improving lipid synthesis and metabolic reprogramming [121]. During energy stress, AMPK activation downregulates ferroptosis by inhibiting lipid peroxidation and maintaining cellular energy balance. This protective mechanism allows tumor cells to survive under metabolic stress conditions [122]. In addition, ferroptosis is downregulated during energy stress, which is mediated by AMPK signaling activation [123]. Conversely, suppressing AMPK/Akt signaling can restore ferroptosis and inhibit the malignancy of CRC [124]. Inhibiting this pathway reduces the survival advantage of cancer cells, increasing their susceptibility to ferroptosis and other forms of cell death. AMPK activation under energetic stress inhibits iron death, which itself, as a lipid peroxidation-driven mode of cell death, is sensitively fine-tuned by multiple lipid metabolic segments (e.g., SCD1 and ACSL4) and signaling pathways (e.g., PI3K/Akt).

### The gut microbiota

The interplay between the gut microbiota and lipid metabolism contributes to the development and metastasis of CRC [125, 126]. A high-fat diet disrupts the homeostasis of the intestinal microbiota, leading to enteritidis and intestinal issues, which significantly increase the risk of CRC [127]. Certain gut microbes and dysregulation of the gut microbiota led to aberrant lipid metabolism. For example, ceramide results in microbiota disorders through the upregulation of TLR4/catenin signaling and the promotion of cholesterol esterification and colorectal tumorigenesis [128]. Secondary bile acids, such as deoxycholic acid (DCA), are produced mainly through microbial metabolism in the gut [129]. Dysregulated secondary bile acids can induce chronic inflammation in the gut, which is a known risk factor for CRC. DCA activates proinflammatory pathways, including the NF-κB and STAT3 pathways, resulting in the production of inflammatory cytokines. These chronic inflammatory components create a tumor-promoting environment [130]. Recently, DCA was shown to promote colorectal carcinoma development by hampering CD8^+^ T-cell function [131]. DCA suppresses the effector functions of CD8^+^ T cells by impairing intracellular calcium accumulation. This disruption in calcium signaling diminishes the activation and cytotoxic activity of CD8^+^ T cells. Moreover, microbiota-derived metabolites can block ferroptosis, facilitating the development of CRC [132]. For example, metabolites such as tyrosol, derived from gut bacteria such as *Faecalibacterium prausnitzii*, have been shown to inhibit ferroptosis in CRC cells. Tyrosol reduces reactive oxygen species (ROS) levels and inflammatory cytokines in tumor cells, thereby preventing the oxidative stress necessary for ferroptosis [133]. With advancements in sequencing techniques and organ culture, the role of the gut microbiota in lipid metabolism and colonic tumors has been thoroughly investigated. Targeting the microbiota might be a novel strategy to improve the prognosis of CRC patients. The gut microbiota and its metabolites (e.g., secondary bile acids and specific metabolites) significantly regulate host lipid metabolism and influence processes such as iron death, thereby participating in the development of CRC. Preclinical evidence supports the feasibility of microbiome-targeted interventions to modulate lipid metabolism in CRC. Fecal microbiota transplantation (FMT) from healthy donors has been shown to restore bile acid homeostasis and reduce secondary bile acid-driven colonic inflammation in murine CRC models [134]. Probiotic supplementation with *Lactobacillus acidophilus* has been reported to attenuate high-fat diet-induced dysbiosis and reduce FASN expression in colonic tissue [135]. Additionally, preclinical and clinical investigations have demonstrated that gut microbiota modulation, including antibiotic preconditioning followed by fecal microbiota transplantation, can reshape the immunological milieu and potentially improve ICI responsiveness by restoring short-chain fatty acid-producing microbial communities [136]. These findings, while preliminary, suggest that microbiome modulation represents a tractable adjuvant strategy for reprogramming lipid metabolism in the CRC TME [136].

### Ferroptosis

As introduced in "SREBPs and SCD" section and "CPT1" section, key lipid metabolism regulators, including SCD1, CPT1, and CD36, are intimately connected to ferroptosis susceptibility. Ferroptosis is an iron-dependent form of programmed cell death characterized by the excessive accumulation of membrane lipid peroxides [137]. Ferroptosis is driven by the failure of the cell’s antioxidant defenses, particularly the activity of the enzyme glutathione peroxidase 4 (GPX4), which normally transforms toxic lipid peroxides into nontoxic lipid alcohols [138]. This failure leads to unchecked lipid peroxidation and eventual cell death [139]. System Xc⁻ is a cystine/glutamate antiporter that plays a crucial role in regulating ferroptosis [137]. It helps maintain redox balance by importing cystine, which is then converted to cysteine and used to produce glutathione (GSH), a key antioxidant that prevents ferroptosis. Iron plays an indispensable role in this process through Fenton chemistry, where iron catalyzes the production of reactive oxygen species (ROS) [140, 141]. ROS attack polyunsaturated fatty acids, leading to lipid peroxidation deposits in the cell membrane [142]. Lipid peroxides disrupt membrane integrity and function, ultimately causing cell death. In CRC, many ferroptosis pathways are mediated through lipid metabolism regulators, such as SCD-1, CPT1, and CD36, suggesting that lipid metabolism is closely related to ferroptosis [91, 143–145]. A recent study demonstrated that the LGR4/Wnt/β-catenin pathway activates the transcription of SLC7A11, a gene that resists ferroptosis. By upregulating SLC7A11, CRC cells can avoid ferroptosis. LRG4 monoclonal antibody treatment effectively blocks the LGR4/Wnt/β-catenin pathway, thereby sensitizing CRC cells to chemotherapy through the promotion of ferroptosis [146]. Ferroptosis represents a novel therapy for CRC, and ferroptosis inducers might serve as adjuncts for the treatment of CRC.

### Noncoding RNAs

**Fig. 2:**
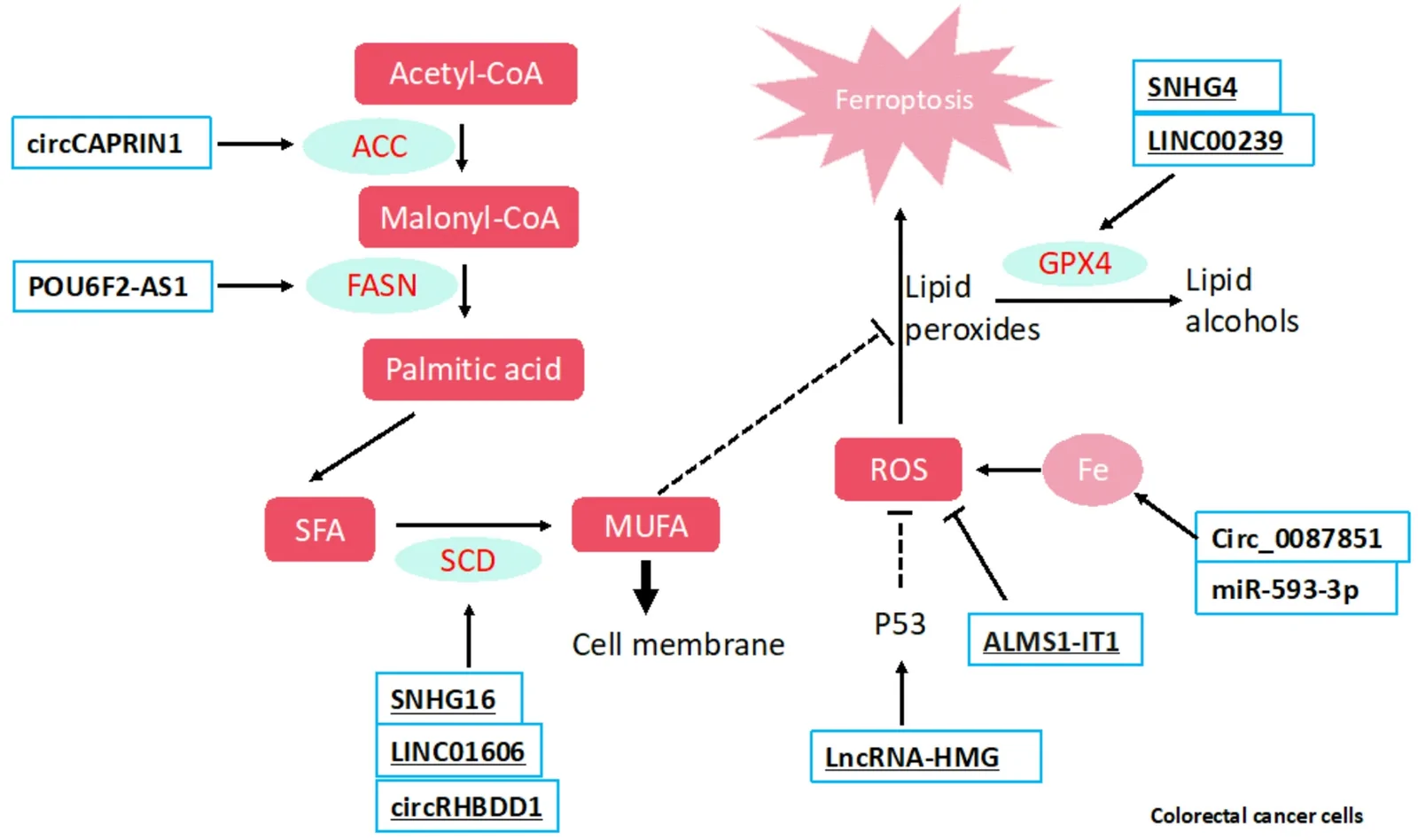
Noncoding RNAs regulate lipid metabolism in colorectal cancer. The blue boxes represent ncRNAs, including circular RNAs (circCAPRIN1, circRHBDD1, and circ-0087851); lncRNAs (POU6F2-AS1, ALMS1-IT1, LncRNA-HMG, LINC00239, LINC01606, and SNHG16); and microRNAs (miRs-593-3p). Noncoding RNAs highlighted in the literature have demonstrated significant potential in protecting colorectal cancer cells from ferroptosis. The dotted lines indicate multiple steps

Noncoding RNAs (ncRNAs), including long noncoding RNAs (lncRNAs) and circular RNAs (circRNAs), are a diverse class of RNA molecules that do not encode proteins but play crucial regulatory roles in various cellular processes [147, 148]. Recent studies have highlighted their involvement in lipid metabolism by influencing lipid synthesis, ferroptosis, and Wnt signaling in colorectal cancer [149–151]. CircRHBDD1 indirectly suppresses ferroptosis by upregulating the expression of SCD, a key enzyme involved in increasing the production of MUFAs, thereby reducing the substrate of lipid peroxidation and facilitating tumor progression [152]. Similarly, LINC01606 protects CRC cells from ferroptosis and enhances cancer stemness through SCD1–Wnt/β-catenin signaling, reinforcing the role of lipid metabolism in tumor survival [153]. Additionally, SNHG16 is associated with Wnt signaling and lipid metabolism, promoting CRC cell survival, migration, and resistance to apoptosis. Silencing SNHG16 reduces cell viability and downregulates genes involved in lipid metabolism, particularly SCD [154]. Conversely, simvastatin enhances antitumor immunity by downregulating PD-L1 expression via the suppression of the expression of the lncRNA SNHG29. SNHG29 stabilizes YAP, a key oncogenic transcription factor that transcriptionally promotes PD-L1 expression, inhibiting immune surveillance [155]. Emerging evidence suggests that ncRNAs regulate ferroptosis by directly modulating ROS production and the expression of key ferroptosis regulators such as GPX4. For example, the knockdown of the lncRNA ALMS1-IT1 exacerbates ferroptosis in CRC, as indicated by reduced GSH/GSSG ratios and increased ROS and iron levels [156]. LncRNA-HMG interacts with p53, promoting its degradation via MDM2, a ubiquitin ligase, thereby bolstering antioxidant defenses and mitigating oxidative stress [157]. Circ_0087851, which is frequently downregulated in CRC, inhibits tumor proliferation and metastasis while inducing ferroptosis by sponging miR-593-3p, restoring the tumor suppressor BAP1. BAP1 enhances ferroptosis sensitivity by promoting iron metabolism and suppressing antioxidant defenses [158]. Additionally, LINC00239 is positively correlated with Nrf2 and GPX4, key regulators of cellular redox homeostasis [159]. Similar roles have been demonstrated for SNHG4 [160]. These findings underscore the pivotal role of ncRNAs in modulating ferroptosis through iron metabolism, redox balance, and oncogenic signaling pathways (Fig. 2). Understanding the interplay among ncRNAs, lipid metabolism, and ferroptosis in CRC could unveil novel biomarkers and therapeutic targets. Targeting ncRNA-mediated pathways may offer a promising strategy to disrupt lipid metabolism and induce ferroptosis, ultimately improving outcomes for CRC patients [161]. Noncoding RNAs profoundly affect CRC progression and iron death sensitivity by regulating lipid metabolism genes and signaling pathways (e.g., SCD1 and Wnt). Ultimately, all these signaling and metabolic alterations converge in the tumor immune microenvironment (TME), affecting the functional status of immune cells and the efficiency of the antitumor immune response.

### Immune microenvironment

Lipid metabolism plays an important role in remodeling the immune microenvironment. The TME is typically immunosuppressive and hypermetabolic. Lipid metabolism shapes these protumorigenic surroundings through various biological processes, including inducing T-cell exhaustion and senescence, preventing cytotoxic cell infiltration, promoting macrophage polarization, increasing Treg differentiation, and causing energy deprivation [162–166]. For example, ACC1, an isoform of ACC, can accelerate Treg differentiation by inhibiting Th17 cell development and promoting an anti-inflammatory response and the formation of an immunosuppressive environment [167]. Cholesterol has been causally linked to CD8⁺ T-cell exhaustion in CRC tissues through endoplasmic reticulum stress (ERS) and mitochondria-mediated energy deprivation, as demonstrated in murine CRC models [168] and supported by correlative human tissue data [169]. The cooperation between lipid metabolism and ERS prevents immune cell infiltration, leading to a worse clinical prognosis [170]. Conversely, intervention in lipid de novo synthesis and ERS can resuscitate T cells and enhance the antitumor response. Treatment with an SCD-1 inhibitor enhances the intratumoral accumulation of CD8^+^ T cells and dendritic cells, which is partly due to decreased ERS [171]. Macrophages and colorectal tumor-resident neutrophils often exhibit M2 or N2 phenotypes, significantly affecting immunotherapy efficacy and the antitumor response [172–174]. Moreover, tumor-resident neutrophils inhibit the infiltration and response of natural killer (NK) cells [175]. Recently, the lipid metabolism-related gene enoyl-CoA-isomerase 2 (ECI2), a mitochondrial enzyme involved in the β-oxidation of unsaturated fatty acids, was shown to inhibit neutrophil extracellular trap (NET) formation, thereby blocking CRC development partially through inhibition of lipid synthesis [176]. Inhibiting the function of SREBP in Treg cells, which is important for lipid metabolism, enhances the antitumor response and significantly decreases tumor size [177]. SREBP-mediated lipid metabolism promotes lactate accumulation and induces dendritic cell tolerance, impeding tumor antigen presentation and response [178]. However, limiting lipid utilization deprives immune cells of energy, leading to cytotoxic T-cell anergy [179]. FASN and lipid synthesis are markedly increased in cancer-associated fibroblasts (CAFs), promoting the accumulation of fatty acids and phospholipids essential for colonic tumor metastasis [180]. The immunosuppressive environment of colorectal tumors, orchestrated by immune and stromal cells, largely depends on lipid metabolism. Targeting lipid metabolism can reverse immunosuppression within the TME, thereby enhancing antitumor immune responses and ICI efficacy [181–186].

It is important to emphasize that lipid metabolic interventions carry inherently cell type-specific consequences and dual, often opposing, effects within the TME. Inhibiting fatty acid synthesis or uptake may simultaneously impair the function of effector CD8⁺ T cells and dendritic cells, which depend on de novo lipogenesis for clonal expansion and antigen presentation [187]. Therefore, the therapeutic window for lipid metabolism targeting requires careful consideration of the delivery strategy, target selectivity, and dosing schedule. Approaches that preferentially act on tumor cells or immunosuppressive populations—such as nanoparticle-formulated FASN inhibitors with enhanced tumor accumulation or CD36 blockade timed to avoid the early T-cell priming phase—may preserve effector immunity while disrupting protumor lipid dependencies. Among the actionable nodes most likely to sensitize mCRC to ICIs without broad immunosuppression are PCSK9 inhibition (which enhances MHC-I recycling on tumor cells), CD36 blockade in Tregs (which selectively impairs their metabolic fitness [52]), and SCD1 inhibition (which reduces endoplasmic reticulum stress-driven T-cell exhaustion while increasing ferroptosis sensitivity).

Collectively, these considerations underscore that targeting lipid metabolism not only directly suppresses tumor growth but also, more importantly, reverses the immunosuppressive TME and enhances antitumor immunity, providing a strong rationale for combination therapy with immune checkpoint inhibitors (ICIs) [52, 187].

#### Lipid metabolic dependencies across CRC molecular subtypes 

The consensus molecular subtypes (CMS) of CRC display distinct lipid metabolic programs that shape both tumor biology and immune microenvironment composition. CMS1 (MSI-immune) tumors exhibit relatively lower de novo lipogenesis but higher immune infiltration, potentially rendering them more susceptible to ferroptosis-inducing strategies that increase existing immune pressure. CMS2 (canonical) tumors are highly dependent on exogenous fatty acid uptake via CD36 and FABPs, making lipid transport blockade a rational therapeutic target. CMS3 (metabolic) tumors are characterized by elevated de novo lipogenesis driven by FASN and SREBPs and show the greatest sensitivity to FASN/ACC inhibition. CMS4 (mesenchymal) tumors upregulate FAO via CPT1, supporting a CAF-rich, immunosuppressive TME with high metastatic potential. The incorporation of CMS-based patient stratification into a lipid metabolism-targeted trial design may improve therapeutic precision and biomarker development.

### Metastatic site-specific lipid–immune niches

The metabolic and immunological landscape of mCRC lesions differs substantially from that of primary tumors and is further shaped by the organ-specific microenvironment at each metastatic site. In liver metastases—the most common site in mCRC—the hepatic lipid-rich milieu promotes CD36-mediated fatty acid uptake by tumor-associated macrophages (TAMs) and resident Kupffer cells, reinforcing M2 polarization and suppressing CD8⁺ T-cell infiltration. Single-cell RNA sequencing of liver metastases revealed a distinct immunosuppressive TAM subset enriched for lipid metabolism genes, including FABP4 and TREM2, which correlated with poor response to immune checkpoint inhibitors (ICIs) [188]. In lung metastases, tumor cells exploit pulmonary surfactant lipids, particularly phosphatidylcholines, to support membrane synthesis and evade oxidative stress, while alveolar macrophages shift toward an anti-inflammatory, lipid-laden phenotype. Peritoneal metastases are characterized by an omentum-derived lipid-supply niche, where cancer-associated adipocytes transfer fatty acids to tumor cells via fatty acid-binding proteins, fuelling β-oxidation and conferring chemoresistance [189]. Across all sites, elevated exogenous lipid availability suppresses effector T-cell function while enhancing Treg and MDSC survival. Recognizing these site-specific lipid dependencies is essential for designing metastatic site-tailored therapeutic strategies.

### Multiomics insights into lipid–immune interactions in mCRC

The integration of single-cell and spatial transcriptomics with lipidomics has begun to generate mechanistic insights that are inaccessible to bulk analyses. Single-cell RNA sequencing of mCRC liver metastases revealed spatially distinct macrophage subsets—particularly *TREM2*⁺/*APOE*⁺/*FABP4*⁺ TAMs—that accumulate in immune-excluded regions and suppress CD8⁺ T-cell function through cholesterol efflux and lipid metabolite signaling [188]. Spatial transcriptomics of primary CRC has mapped the colocalization of lipid synthesis gene programs (FASN and SCD1) with exhausted T-cell signatures at the tumor–stroma interface, suggesting that lipid metabolic gradients establish spatially defined immunosuppressive niches [190]. Metabolomic and lipidomic profiling of matched primary and mCRC specimens has revealed site-specific lipid signatures—including elevated lysophosphatidylcholines in liver metastases and increased sphingolipid species in peritoneal deposits—that may serve as biomarkers of metastatic niche adaptation [12]. Collectively, these multiomics studies provide a high-resolution landscape of lipid-driven immune remodeling in mCRC and identify actionable metabolic nodes with spatial and cell type specificity.

## Lipid metabolism–related therapeutic strategies for colorectal cancer

### Lipid metabolism-based therapy in CRC

**Fig. 3:**
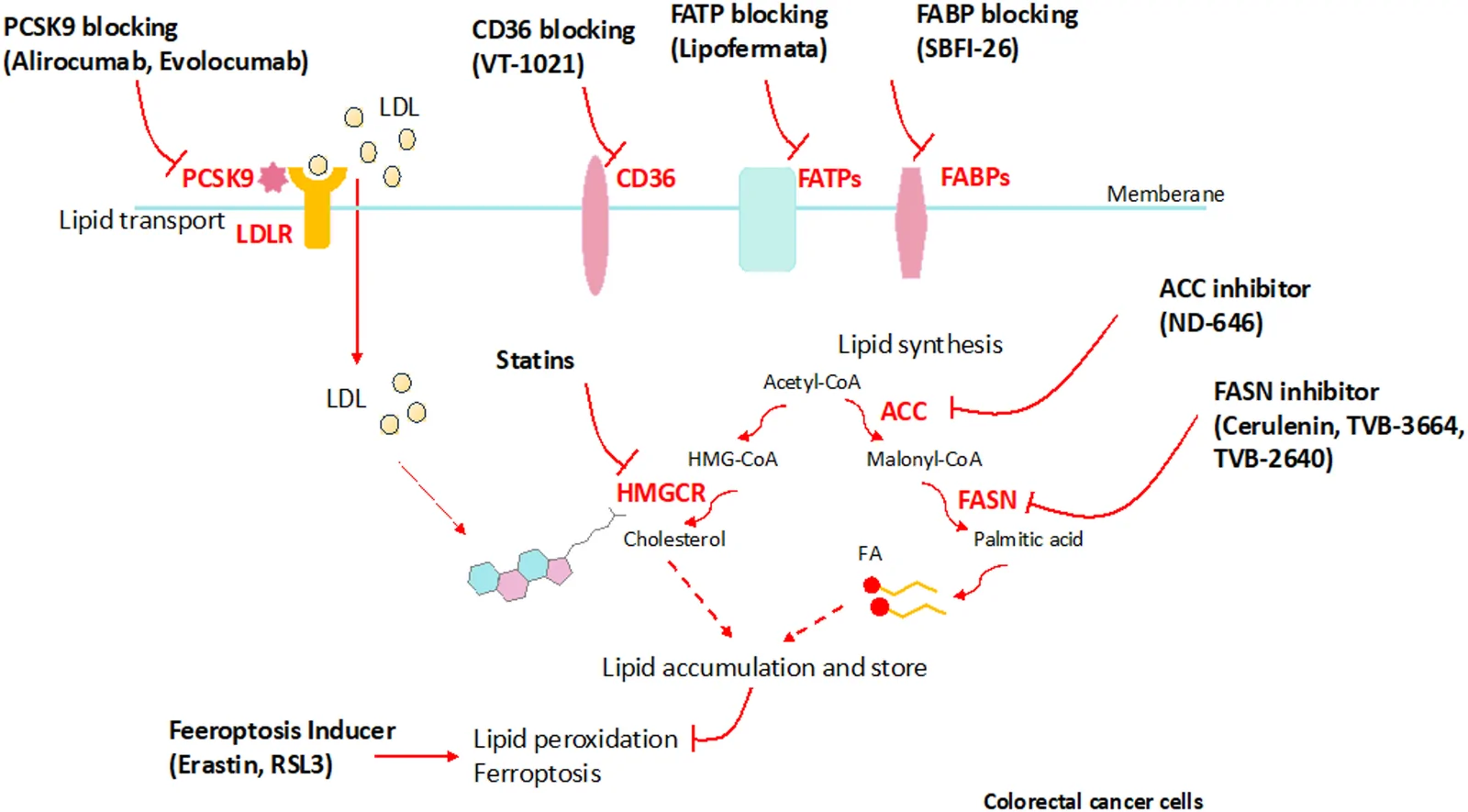
Lipid metabolic reprogramming and therapeutic strategies in colorectal cancer. Enhanced lipid metabolism in colorectal cancer cells occurs through increased lipid synthesis and transport to sustain increased biological activity. Lipid synthesis originates from acetyl-CoA, leading to the production of fatty acids and cholesterol, which is catalyzed by key enzymes such as ACC, FASN, and HMGCR. Additionally, with the assistance of PCSK9, abnormally expressed LDLR enhances LDL absorption and transmembrane transport, promoting LDL cholesterol-mediated colorectal cancer progression. Increased lipid synthesis facilitates lipid accumulation and storage, preventing lipid peroxidation and Fe-mediated ferroptosis. Blocking fatty acid synthesis by inhibiting ACC and FASN has been extensively studied in preclinical research. Statins and PCSK9 inhibitors are currently the most effective drugs for lowering cholesterol levels in clinical settings. Furthermore, blocking lipid transport proteins such as CD36, FATPs, and FABPs has attracted significant research interest as a potential therapeutic approach. The dotted lines represent multiple steps. These therapeutic strategies span multiple stages of clinical development. Preclinical-stage agents include C75, C93, orlistat, Fasnall, TVB-3664, Soraphen A, TOFA, ND-646, FA6-152, JC63.1, ONA-046, Lipofermata, Grassofermata, BMS309403, SBFI-26, HTS01037, erastin, RSL3, FIN56, ML210, evolocumab, and alirocumab (in oncology). Early-phase clinical agents include TVB-2640 (Phase I/II), CPI-613 (Phase II), EGCG (Phase II), sulfasalazine (Phase II), artemisinin (Phase I/II), and VT1021 (Phase I). Sorafenib is FDA approved for hepatocellular and renal cell carcinoma and is under investigation in CRC. Statins and PCSK9 inhibitors (alirocumab, evolocumab, and inclisiran) are approved for cardiovascular indications but remain investigational in CRC. Notably, no lipid metabolism-targeting agent is currently approved specifically for CRC

On the basis of a deeper understanding of the mechanisms underlying the reprogramming of lipid metabolism in CRC and its interaction with the immune microenvironment (as previously described), targeting this process has emerged as a highly promising therapeutic strategy. This section systematically reviews current advances in targeted therapies according to the metabolic segments involved (synthesis, transport, catabolism/oxidation, and specific modes of death), with a focus on combination strategies with immunotherapy (Fig. 3). Inhibitors of key enzymes involved in lipid biogenesis, such as FASN and ACC, have shown promising results in various tumor models [191–193]. However, interfering with cholesterol metabolism through statins and PCSK9 inhibitors has been demonstrated to inhibit tumor progression in some preclinical models [194–198]. Inhibiting lipid transport proteins, such as CD36, could significantly disrupt the energy homeostasis and biological activities of tumor cells, making these ideal targets for tumor therapy [199–201]. Importantly, immune checkpoint inhibition has revolutionized cancer therapy. Combining this approach with therapies targeting lipid metabolism might greatly improve the treatment of CRC patients.

### Targeting fatty acid synthesis

Targeting lipid synthesis in CRC is a promising strategy, as tumor cells often exhibit increased lipogenesis to support their biological activities. Many studies have shown that inhibiting fatty acid synthesis by targeting FASN and ACC significantly impedes CRC development [202–204]. Various FASN inhibitors, such as C75, C93, Fasnall, and epigallocatechin gallate (EGCG), have shown promising results in reducing lipid synthesis and inhibiting tumor growth (Table 1) [205–209]. Some FASN inhibitors, including cerulenin, TVB-3664, and orlistat, have exhibited promising inhibitory effects on CRC cell proliferation (Table 1). Cerulenin induces apoptosis in CRC cell lines and has been shown to reduce liver metastasis when used in combination with oxaliplatin [210, 211]. TVB-3664 has demonstrated strong antitumor effects in various tumor models, including CRC. The inhibition of FASN by TVB-3664 in colorectal cancer models leads to the selective upregulation of the expression of the fatty acid transporter CD36, which compensates for the loss of FASN activity. Combining the FASN inhibitor TVB-3664 with the CD36 inhibitor sulfosuccinimidyl oleate (SSO), an irreversible inhibitor that blocks the uptake of fatty acids, synergistically reduces cell proliferation and tumor growth. To date, TVB-2640 is the only FASN inhibitor currently being assessed for safety and efficacy in clinical trials. In a phase I clinical trial, TVB-2640 demonstrated significant FASN inhibition potential with manageable side effects. The treatment shows promising disease control rates (DCRs) in solid tumors (42% as monotherapy), and the DCR is greatly improved (70%) when it is combined with paclitaxel [208]. A window-of-opportunity trial (NCT02980029) evaluating TVB-2640 in resectable colon cancer was terminated because of suboptimal patient enrollment rather than safety signals or a lack of biological activity. This underscores a practical challenge in early-phase CRC trials involving lipid metabolism inhibitors: the requirement for presurgical biopsy windows and stringent eligibility criteria often limit accrual. Future trial designs should incorporate adaptive enrollment strategies and biomarker-driven patient selection to improve feasibility. Encouragingly, a phase II study in astrocytoma patients confirmed the safety and efficacy of combining TVB-2640 with bevacizumab, successfully meeting its primary endpoint [212]. Additionally, phase IIa trial data revealed positive results of TVB-2640 in reducing liver fat content and ameliorating inflammation, highlighting its therapeutic potential in addressing lipid metabolism dysregulation and fatty liver disease [213]. In addition to inhibiting CRC, FASN inhibitors have shown favorable results in inhibiting breast cancer, prostate cancer, and lung cancer [209, 214, 215].

ACC is a key component in FASN-mediated fatty acid synthesis. Blocking ACC greatly reduces fatty acid synthesis efficiency, making it a potential target for tumor therapy. The downregulation of ACC expression reliably suppresses lipogenesis and tumor development in vivo [192, 216–219]. Inhibitors such as soraphen A, CPI-613, ND-654, and TOFA have shown promising results in reducing tumor development through the inhibition of lipid synthesis (Table 1) [220–222]. TOFA significantly inhibited fatty acid synthesis in colon carcinoma cells. This inhibition leads to apoptosis, making TOFA a promising compound for potential cancer treatments [223]. Moreover, treatment with an ACC inhibitor has been shown to significantly decrease long-chain fatty acid synthesis in breast cancer cells [224]. Similar phenotypes were demonstrated in non-small cell lung cancer (NSCLC) patients after treatment with the ACC inhibitor ND-646 [225]. ACC inhibitors are also being explored for their potential in treating obesity and metabolic diseases such as type II diabetes mellitus (T2DM) and nonalcoholic steatohepatitis (NASH) [226, 227] (Table 2).

**Table 1:** Lipid metabolism-based therapies for CRC

Therapy	Mechanisms, functions and applications	Clinical Trials.gov ID	Stage of Development	Relevant Cancer Type (if not CRC)
C75	C75 is a synthetic inhibitor known for its anticancer properties. It is also a potent activator of CPT1A.	None	Preclinical	Multiple
C93	C93 has shown promising anticancer activity through inducing apoptosis.	None	Preclinical	Multiple
Orlistat	Orlistat, a weight-loss drug, also irreversibly inhibits FASN, showing anticancer effects by targeting its thioesterase domain. This results in reduced tumor cell proliferation, apoptosis induction, and decreased tumor growth in colorectal cancer models.	None	Preclinical	Multiple
EGCG	Epigallocatechin gallate (EGCG), a natural polyphenol in green tea. EGCG has shown great potential in inhibiting cell proliferation and inducing apoptosis in CRC cell lines.	NCT02891538	Phase II (NCT02891538)	Multiple
Fasnall	Fasnall, a thiophenopyrimidine compound, effectively inhibits cancer cell growth by blocking fatty acid synthesis.	None	Preclinical	Breast (HER2+)
Cerulenin	Cerulenin is a natural antifungal antibiotic derived from the fungus *Cephalosporium caerulens*. Cerulenin inhibits fatty acid and sterol biosynthesis by binding to β-ketoacyl-ACP synthase. In preclinic models, cerulenin induced apoptosis and reduced liver metastasis in CRC tumor models.	None	Preclinical	Multiple
TVB-3664	TVB-3664, an orally available FASN inhibitor, has exhibited promising inhibition efficiency in CRC cell proliferation. Combining the FASN inhibitor TVB-3664 with the CD36 inhibitor blocks the uptake of fatty acids, synergistically reduces cell proliferation and colon tumor growth in vivo.	None	Preclinical	Multiple
TVB-2640	TVB-2640 (Denifanstat) has demonstrated significant FASN inhibition potential with manageable side effects in clinical trials. TVB-2640 (Denifanstat) is the most clinically advanced FASN inhibitor, having entered Phase I/II trials in solid tumors. A window-of-opportunity trial in resectable colon cancer (NCT02980029) was terminated due to enrollment challenges rather than safety concerns. Phase II data in astrocytoma and early-phase data in breast cancer support its biological activity.	NCT02980029	Phase II (astrocytoma); Phase I (solid tumors)	Breast, astrocytoma
Soraphen A	Soraphen A is a myxobacterial metabolite known for its potent inhibitory effects on ACC.	None	Preclinical	Multiple
CPI-613	CPI-613 (devimistat) disrupts lipid metabolism by targeting ACC. This inhibition is mediated through the activation of AMPK, which in turn inactivates ACC.	NCT05733000	Phase II (NCT05733000)	Pancreatic, AML
TOFA	TOFA (5-(Tetradecyloxy)-2-furoic acid), a potent inhibitor of ACC1, mainly induces cell cycle arrest and apoptosis by inhibiting the PI3K/Akt/mTOR pathway.	None	Preclinical	Multiple
ND-646	ND-646 is a potent, orally bioavailable allosteric inhibitor of ACC1 and ACC2. This inhibition leads to reduced tumor growth and increased apoptosis.	None	Preclinical	NSCLC
FA6-152	FA6-152 is a monoclonal antibody that targets CD36. It has been widely used in research to study the role of CD36 in cancer.	None	Preclinical (research tool)	Multiple
JC63.1	JC63.1 is a monoclonal antibody that specifically targets CD36.	None	Preclinical (research tool)	Multiple
VT1021	VT1021 is a novel immuno-oncology agent. It targets both CD36 and CD47, playing a dual role in cancer therapy. By blocking the CD47 immune checkpoint and modulating CD36 signaling through thrombospondin-1 mimicry, VT1021 induces tumor cell apoptosis and inhibits angiogenesis.	NCT03364400	Phase I (NCT03364400)	Multiple solid tumors
ONA-046	ONA-046 is an antibody drug targeting cells that initiate metastasis in cancer. It works by blocking fat intake in cancer cells. Preclinical studies have shown that ONA-046 can significantly reduce tumor growth and metastasis.	None	Preclinical	Multiple
Lipofermata	Lipofermata is a potent inhibitor of FATP2. It has shown significant efficacy in blocking the uptake of LCFAs and vLCFAs.	None	Preclinical	Multiple
Grassofermata	Grassofermata is a potent inhibitor of FATP2. It effectively blocks the uptake of fatty acids in various cell types.	None	Preclinical	Multiple
DCA	Deoxycholic acid (DCA), a secondary bile acid, contribute to the emulsification and absorption of fats in the intestine. DCA is also studied for its potential to inhibit FATP5,	None	Preclinical	—
UDCA	Ursodeoxycholic acid (UDCA) is another bile acid that inhibits FATP5. Research has shown that UDCA can effectively reduce the uptake of LCFAs in liver.	NCT00062023	Phase II/III (NCT00062023)	Liver disease (approved)
BMS309403	BMS309403 is a potent and selective inhibitor of FABP4. This compound also shows some activity against FABP3 and FABP5, but with much lower affinity. It is being explored for its role in reducing tumor growth in cancer research.	None	Preclinical	Multiple
SBFI-26	SBFI-26 is a selective inhibitor of FABP5 and FABP7. It has been explored for its anti-tumor properties.	None	Preclinical	Prostate
HTS01037	HTS01037 shows a high-affinity antagonist of FABP4. This compound also shows some activity against other FABPs, such as FABP3, FABP5, and FABP7, but with lower affinity.	None	Preclinical	Multiple
Mupirocin	Mupirocin has been identified as a novel inhibitor of fat mass and obesity-associated protein (FTO), an enzyme that regulates mRNA methylation. By inhibiting FTO, mupirocin induces ferroptosis in CRC. This leads to reduced tumor growth and enhanced sensitivity to ferroptosis inducers like erastin and RSL3.	None	Preclinical	Multiple
Sulfasalazine	Sulfasalazine induces ferroptosis in cancer cells by inhibiting the system Xc- and increasing ROS production. It has shown great potential in suppressing CRC development and metastasis. In addition, Sulfasalazine sensitizes colorectal cancer to radiotherapy.	NCT06134388	Phase II (NCT06134388)	IBD (approved)
FIN56	FIN56 is a specific inducer of ferroptosis. It operates through two distinct pathways: degrading GPX4 and activating squalene synthase. FIN56 treatment promotes the efficacy of oxaliplatin to overcome the resistance in CRC.	None	Preclinical (research tool)	Multiple
ML210	ML210 is a selective and covalent inhibitor of GPX4. ML210 has shown anticancer activity, particularly in cells expressing mutant *Ras*.	None	Preclinical (research tool)	Multiple
Artemisinin	Artemisinin shows potential in inducing ferroptosis. In cancer treatment, artemisinin can sensitize colorectal cancer cells to ferroptosis, enhancing the effectiveness of cancer therapies by promoting cell death.	NCT02633098	Phase I/II (NCT02633098)	Multiple
Sorafenib	Sorafenib has been found to induce ferroptosis. Research indicates that sorafenib’s ability to induce ferroptosis involves the accumulation of intracellular iron and ROS, which disrupts cellular metabolism and leads to CRC death.	NCT00326495	FDA-approved (HCC, RCC); Phase II in CRC	HCC, RCC
Erastin	Erastin is a small molecule. It induces ferroptosis by inhibiting system Xc-. Several reports have demonstrated that erastin induces ferroptosis in CRC.	None	Preclinical (research tool)	—
RSL3	RSL3 is a compound known for its role as a ferroptosis activator. It works by inhibiting GPX4. Several reports have demonstrated that RSL3 induces ferroptosis in CRC	None	Preclinical (research tool)	—
Alirocumab	Alirocumab is a monoclonal antibody used to lower LDL cholesterol levels.	None	Preclinical in oncology	CVD (approved)
Evolocumab	Evolocumab is designed to bind to PCSK9 and inhibit PCSK9 from binding to LDL receptors on the liver surface.	None	Preclinical in oncology	CVD (approved)
Inclisiran	Inclisiran is a siRNA medication used to lower LDL cholesterol levels.	None	FDA-approved (CVD); Preclinical in oncology	CVD (approved)
Statins	Statins, are drugs used to lower cholesterol levels by inhibiting the enzyme HMG-CoA reductase. Statins are widely used as cholesterol-lowering agents in cardiovascular disease. Preclinical and observational data suggest potential antitumor activity in CRC; however, statins have not been approved for CRC treatment, and clinical trial results remain inconsistent.	NCT01011478	Observational/retrospective in CRC	CVD (approved)

**Table 2:** Key studies linking lipid metabolic alterations to immune remodeling in CRC and metastases

Study (PMID)	Model	Key lipid marker	Immune readout	Intervention	Evidence type
PMID: 28854168	Mouse/Human CRC	CD36	CD8⁺ T-cell exhaustion, Treg uptake	CD36 blockade	Causal (in vivo)
PMID: 33125861	Human liver metastasis	FABP4, TREM2 macrophages	TAM immunosuppression, ICI resistance	None	Correlative (scRNA-seq)
PMID: 31217590	Mouse CRC	Cholesterol	CD8⁺ T-cell exhaustion via ERS	Statin	Causal (in vivo)
PMID: 34715016	Human CRC cohort	FASN	Treg/CD8 ratio, PD-L1	FASN inhibitor	Correlative + preclinical
PMID: 30736843	Human CRC/metastasis	Lysophosphatidylcholines	NK cell exclusion	None	Correlative (lipidomics)

### Targeting cholesterol metabolism

Upregulated cholesterol metabolism is closely associated with CRC development [228, 229]. Statins and PCSK9 inhibitors are two key methods that affect cholesterol metabolism. Targeting cholesterol-mediated signaling could directly block cholesterol utilization by tumor cells and impede their high biological activity. Moreover, it might reverse tolerance and the protumor environment by remodeling the immune system [230, 231]. Research has indicated that statins may increase the efficacy of chemotherapy by sensitizing CRC cells to treatment, potentially overcoming drug resistance [232, 233]. This sensitization occurs because statins can induce apoptosis and inhibit cell proliferation, increasing the susceptibility of cancer cells to chemotherapeutic agents. Additionally, the anti-inflammatory properties of statins may contribute to their anticancer effects. Clinical studies on the effectiveness of statins in treating CRC have yielded mixed results [44, 234]. Some suggest a modest protective effect, with statin users showing a lower incidence of CRC and improved survival rates [235–237]. In a phase II study (NCT01281761), the combination of simvastatin and cetuximab/irinotecan demonstrated promising results in *Kras*-mutant CRC patients resistant to cetuximab/irinotecan-based chemotherapy [238]. However, others have reported no significant impact on cancer incidence or mortality [239, 240]. For example, in a phase II trial, simvastatin was not able to restore sensitivity to cetuximab in CRC patients with *Kras* mutations [239]. Despite the mixed clinical data, the potential benefits of statins in CRC treatment warrant further investigation. Ongoing research aims to better understand the underlying mechanisms through which statins exert their antitumor effects.

PCSK9 plays multifaceted roles in colorectal cancer by regulating signaling pathways associated with cholesterol metabolism [241–243]. A PCSK9 inhibitor can specifically inhibit LDLRs, impeding the transportation and utilization of cholesterol. Targeting PCSK9 could improve CRC treatment outcomes by both directly inhibiting tumor growth and enhancing immune response ability [78, 84]. A phase II trial (NCT06391905) is currently investigating the combination of a PCSK9 inhibitor with standard first-line treatments in patients with advanced colorectal cancer with proficient mismatch repair (pMMR) and microsatellite stable (MSS) status; the specific agent and primary endpoints have not yet been publicly disclosed. The outcomes of these clinical trials will provide valuable insights into the efficacy and safety of PCSK9 inhibition as a therapeutic strategy for colorectal cancer. PCSK9 inhibitors have been widely used in cardiovascular disease but not yet extensively in CRC or other tumors [44, 244, 245]. Alirocumab and evolocumab (Table 1) are two monoclonal antibodies that specifically inhibit PCSK9 and have been approved by the FDA [75, 246]. Small-molecule inhibitors, such as BMS-962,476, can specifically block the activity of PCSK9 [247]. Inhibiting LDLR through targeting of PCSK9 can markedly lower LDL cholesterol levels and is widely applied in cardiovascular diseases [248]. Recent research has focused on identifying small-molecule inhibitors that can disrupt the PCSK9–LDLR interaction. For example, studies have shown that drugs such as benazepril and quinapril exhibit high potency as PCSK9-LDLR disruptors, suggesting their potential as lipid-lowering agents for treating cancer and cardiovascular diseases [249–251].

### Targeting lipid transport proteins

Targeting lipid transport proteins can disrupt lipid metabolism and energy homeostasis in tumor cells. CD36, FATPs, and FABPs are the most prevalently expressed and upregulated lipid transport proteins during tumor development, with CD36 being the most extensively studied protein [199, 201, 252]. Accumulating data demonstrate that blocking CD36 with antibodies or small molecule inhibitors can inhibit tumor growth and metastasis (Table 1) [253–255]. Despite these promising preclinical findings, there are currently no clinical trials specifically targeting CD36 inhibition in patients with colorectal cancer. VT1021 is an investigational agent targeting thrombospondin-1 signaling; early-phase clinical data have demonstrated preliminary antitumor activity, although its role in CRC remains to be established in controlled trials [256]. Ongoing research and future clinical studies will be crucial for determining the efficacy and safety of CD36-targeted therapies in patients with colorectal cancer [211].

Blocking lipid transport could be a promising strategy for treating metabolic diseases and cancers. Lipofermata and Grassofermata are notable inhibitors of FATPs and effectively limit lipid transport and tumor growth [257–261]. BMS309403 and HTS01037 target FABP4 [262, 263], whereas SBFI-26 targets FABP5 (Table 1) [264]. All of these compounds have shown potential in the treatment of lipid metabolic diseases [265, 266]. Although their role in lipid metabolism suggests that they could be potential therapeutic targets, research on FATPs and FABPs in CRC is limited.

### Targeting ferroptosis in CRC

Aberrant ferroptosis is closely related to dysregulated lipid metabolism and tumor diseases [267], making it a promising target for tumor therapy [268]. Recent studies have shown that inducing ferroptosis can promote CRC cell death and help overcome resistance issues in cancer treatment [269–271]. By blocking GPX4 and System Xc⁻, researchers aim to increase oxidative stress in cancer cells, leading to their death and potentially improving the effectiveness of existing cancer treatments. Ferroptosis inducers (Table 1) inhibit CRC growth. For example, RSL3 directly inhibits GPX4, causing an increase in lipid peroxides and inducing ferroptosis in CRC cells. RSL3-induced ferroptosis can be enhanced by the use of cetuximab, an epidermal growth factor receptor (EGFR) inhibitor, for CRC treatment in the clinic [272]. Preclinical studies have demonstrated the efficacy of RSL3 in eliminating CRC cells both in vitro and in xenograft models [273, 274]. Erastin inhibits system Xc-, leading to the depletion of intracellular GSH. This depletion results in the accumulation of lipid peroxides and subsequent ferroptosis in CRC cells [275]. We have summarized other ferroptosis inducers applied in other tumor treatments in Table 1. The use of ferroptosis inducers alone or in combination with existing therapies can increase treatment efficacy and reduce resistance in CRC.

It is important to distinguish between ferroptosis inducers suitable for clinical translation and those used primarily as research tools. Erastin and RSL3 are prototypical research-grade compounds with significant systemic toxicity and suboptimal pharmacokinetic profiles that currently preclude direct clinical application. In contrast, sorafenib and sulfasalazine are clinically approved or actively investigated agents with established safety records; their ability to induce ferroptosis may contribute to their antitumor activity in CRC, although this mechanism has not been confirmed as primary in clinical settings. Artemisinin and its derivatives represent an intermediate category, with early-phase trials exploring their ferroptosis-related efficacy. A key challenge for translating ferroptosis induction into clinical practice is achieving tumor-selective delivery while minimizing systemic oxidative toxicity; nanoparticle-based and prodrug formulation strategies are under active investigation to address this barrier [268, 270].

### Combination therapy in CRC

In view of the close connection between lipid metabolism reprogramming and the immunosuppressive TME, combining drugs that target lipid metabolism with ICIs can theoretically simultaneously combat tumor cells and deregulate immunosuppression, achieving synergistic efficacy and overcoming drug resistance. This strategy is a popular and important direction of current research. Immune checkpoint inhibitors targeting PD-1/PD-L1 and CTLA-4 have made great progress in tumor therapy. Nivolumab, dostarlimab, and pembrolizumab have been widely used in the clinic and have significantly improved the survival of CRC patients [276–278]. However, the therapeutic efficacy for treating CRC remains modest and is often accompanied by drug resistance [279–281]. Combining immune checkpoint inhibitors (ICIs) with lipid metabolism-based therapies offers a promising new approach for CRC treatment, potentially improving antitumor efficiency and overcoming resistance issues. Immune checkpoint inhibition can remodel the immune microenvironment and enhance the cytotoxic cell response. Moreover, lipid metabolism-based therapies, including those that block lipid synthesis, intervene in lipid transport and utilization, and induce ferroptosis, can disrupt tumor cell homeostasis.

The combination of PD-1 antibodies and PCSK9 inhibition has been widely studied in various cancers [83, 282, 283]. In CRC, PCSK9 inhibition has been reported to significantly increase the antitumor effect of anti-PD-1 therapy [84]. Fumarate hydratase (FH), an enzyme that converts fumarate to malate in the citric acid cycle, is positively related to CRC prognosis and has been demonstrated to block the transcription of PCSK9. This blockade increases the clonal expansion of CD8^+^ T cells. FH treatment combined with an anti-PD-1 antibody effectively improves antitumor efficacy [284]. In addition, in vivo data have shown that anti-PD-1 therapy promotes the expression of PCSK9 and CD36 and modestly inhibits CRC development. The combination blockade of PCSK9 and PD-1 elicits a synergistic effect, suggesting that PCSK9 inhibitors could improve the efficacy of ICIs.

Combining ferroptosis inducers with existing therapies can increase treatment efficacy and reduce resistance in CRC. Ubiquitin-specific protease 8 (USP8) deubiquitinates GPX4 and protects it from degradation by the proteasome. Blocking USP8 destabilizes GPX4 and sensitizes cells to ferroptosis. Combining a USP8 inhibitor with a ferroptosis inducer enhances the clonal expansion of CD8^+^ T cells and impedes colorectal tumor growth, thereby enhancing the antitumor response to PD-1 immunotherapy in vivo [285]. Conversely, the enzyme CYP1B1 promotes resistance to ferroptosis in CRC. CYP1B1 achieves this by degrading ACSL4, an enzyme essential for ferroptosis, through the activation of the protein kinase C pathway [286]. This degradation process helps CRC cells evade ferroptosis, contributing to tumor survival and progression. Inhibiting CYP1B1 in combination with PD-1 antibodies greatly improves the immune response to tumors [287]. In conclusion, combining immune checkpoint inhibition with ferroptosis-dependent cell death in CRC could improve the tumor response and overcome resistance issues [287–289].

## Research limitations and future directions

Although lipid-lowering treatments are well recognized in cardiovascular medicine, their application in CRC oncology is still in its early stages, yielding inconsistent or limited clinical outcomes. The study of statin and FASN inhibitor trials, particularly TVB-2640, is pertinent to colorectal cancer research. Further exploration is necessary to elucidate the pathways connecting specific lipid metabolic nodes to oncogenic and immune effects in colorectal cancer. Lipidomics, which primarily employs mass spectrometry (MS), faces challenges in terms of comprehensive coverage, sensitivity, and resolution, particularly for low-abundance or isomeric lipids essential for membrane dynamics and signaling. To achieve real-time in vivo lipid metabolism tracking, metabolic flux analysis must be refined. This complexity is heightened by oncogenic mutations such as those in KRAS or BRAF. Given heterogeneity, stratified therapeutic approaches are imperative.

Future research should emphasize several core directions. First, CRISPR/Cas9 functional genomics screens and integrated multiomics strategies—encompassing genomics, transcriptomics, proteomics, lipidomics, and metabolomics—combined with artificial intelligence should be employed to delineate the complex regulatory networks of lipid metabolism in CRC and identify novel master regulators. Epigenetic regulators such as KMT2A warrant further investigation as potential predictive biomarkers. Second, the development and application of cutting-edge lipidomics platforms—including ion mobility spectrometry–mass spectrometry (IMS–MS)–--should be prioritized to achieve enhanced sensitivity, resolution, and coverage in the comprehensive characterization of the CRC lipidome and the identification of clinically actionable lipid biomarkers. Third, enumerating lipid metabolic vulnerabilities, particularly those associated with CRC molecular subtypes (CMSs) and oncogenic mutations (e.g., KRAS mutations), facilitates the implementation of personalized medicine strategies. The identification of biomarkers for predicting the response to lipid metabolism inhibitors is encompassed within this scope. Finally, combining advanced lipidomics for patient stratification, interpreting subtype-specific dependencies, and engineering intelligent combination therapies aimed at lipid metabolism in conjunction with immune and cell death pathways are crucial. These initiatives are designed to transform the extensive mechanistic insights detailed in this review into concrete improvements in the prognosis and treatment of patients with colorectal cancer.

## Conclusion

Lipid metabolic reprogramming is a fundamental driver, rather than a bystander, of CRC progression. CRC cells increase de novo lipogenesis, cholesterol biosynthesis, fatty acid uptake, and oxidative modification to supply the energy, structural components, and signaling molecules required for sustained proliferation, invasion, metastasis, and therapeutic resistance. Dysregulated Wnt/β-catenin and PI3K/Akt pathways form positive feedback loops with lipid metabolism, further promoting tumor growth and survival. In addition to tumor cells, lipid metabolic remodeling shapes an immunosuppressive tumor microenvironment by promoting T-cell exhaustion, enhancing Treg function, inducing M2 macrophage polarization, and fostering dendritic cell tolerance. Interactions between the gut microbiota and host lipid metabolism influence ferroptosis sensitivity, with PUFA levels and lipid peroxidation-related redox balance constituting a key regulatory axis. On the basis of these insights—although much evidence remains correlative or preclinical—targeting lipid metabolism has emerged as a promising therapeutic strategy, encompassing the inhibition of lipid synthesis enzymes, transporters, and cholesterol regulators and the induction of ferroptosis. Combination therapies integrating lipid metabolism modulators with immune checkpoint inhibitors have demonstrated synergistic antitumor effects in preclinical models, with early-phase clinical investigations ongoing; rigorous randomized trials are needed to validate these strategies in patients.

## Data Availability

Not applicable.
